# Gender differences in plaque characteristics of nonculprit lesions in patients with coronary artery disease

**DOI:** 10.1186/s12872-019-1023-5

**Published:** 2019-02-26

**Authors:** Jiangtian Tian, Xuedong Wang, Jinwei Tian, Bo Yu

**Affiliations:** 1 Key Laboratory of Myocardial Ischemia, Chinese Ministry of Education, Harbin, China; 2 Department of Cardiology, The Second Affiliated Hospital of Harbin Medical University, Harbin, 150086 China

**Keywords:** Coronary artery disease, Plaque characteristic, Gender, Optical coherence tomography, Nonculprit plaque

## Abstract

**Background:**

Although numerous reports suggest sex-related differences in atherosclerosis, limited data describing gender-associated differences in plaque morphology and composition are currently available. The aim of the present study was to compare coronary nonculprit plaque characteristics in women and men with coronary artery disease (CAD) by optical coherence tomography (OCT).

**Methods:**

This was a retrospective study. A total of 187 nonculprit plaques were identified in 103 patients with CAD who underwent OCT imaging of all 3 coronary arteries. These patients included 77 (74.8%) men and 26 (25.2%) women.

**Results:**

Female patients were significantly older than males (mean age, 70.8 ± 7.3 vs 60.8 ± 9.8 years; *P* < 0.001) and less likely to be current smokers (*P* = 0.007). OCT analysis included the presence of lipid-rich plaque, maximum lipid arc, lipid-core length, lipid index (LI), fibrous cap thickness, and the incidence of thin-cap fibroatheroma (TCFA). Nonculprit plaques in men exhibited greater lipid-core length and LI compared with those of women (9.4 ± 4.5 vs. 7.3 ± 4.3 mm, *P* = 0.024; 1615.1 ± 893.8 vs. 1237.8 ± 859.8, *P* = 0.035, respectively). In the univariate linear regression model, sex and current smoker were all associated with a larger LI, whereas only use of statin was independent risk factor for a larger LI in multivariate analysis.

**Conclusions:**

Coronary nonculprit plaques in male patients with CAD contain larger lipid cores than those of female patients.

## Background

Coronary artery disease (CAD) is the most common cardiovascular disease caused by coronary stenosis, spasm or occlusion. Despite widespread use of established medical therapies, CAD remains the leading cause of mortality in women in most developed countries. It is estimated that up to 23.6 million people will succumb to cardiovascular disease by 2030 [1] because of increasing obesity, whose rate has doubled between 1980 and 2008, and is expected to further increase.

Epidemiological studies have shown that males are more affected by accelerated atherosclerotic cardiovascular disease (CVD) compared with women [2–4]. The discovery of sex-dependent disparities in the development of cardiovascular disease has prompted studies aiming at further understanding of ischemic heart disease in women along the continuum of clinical care. In general, the first CVD event occurs 9 years earlier in males compared with females, although this gap narrows with age [5]. The reasons for gender differences in CVD are complex and include clinical risk profile, downstream effects of sex hormones and behavior, although they cannot in many ways fully account for outcome disparity [6–8].

Although women are thought to have a greater symptom burden and a higher rate of functional disability but a lower prevalence of obstructive CAD [9–11], plaque morphology and composition data comparing coronary artery disease in women and men are scarce. A few pathological studies suggested that gender may confer differences in coronary plaque characteristics [12]. These findings indicate a potentially distinct form of pathophysiology for atherosclerosis in women. The underlying pathophysiology is probably related to the plaque type. Studies have reported that ruptured and/or vulnerable plaques exist at nonculprit lesions, as well as in culprit segments in acute coronary syndrome (ACS) patients [13–15]. Moreover, patients with ACS that underwent percutaneous coronary intervention were shown to have similar recurrent adverse cardiovascular event rates in culprit and nonculprit lesions (12.9% versus 11.6% during a 3-year follow-up period) [16]. However, the characteristics of nonculprit plaques in CAD patients have not been clearly defined. Therefore, to compare coronary nonculprit plaque characteristics in CAD patients in vivo between genders may help understand the underlying pathophysiology of CAD and prevent recurrent cardiovascular events.

Optical coherence tomography (OCT) is an intravascular imaging tool that allows visualization of coronary arteries with high resolution [17]. OCT provides detailed structural information on intracoronary pathology, including plaque morphology, components, and microstructures associated with instability in vivo. The OCT characteristics for various components of atheromatous plaques have been validated in a histology-controlled study [18] As such, the technique provides a unique platform to evaluate the association of gender with coronary artery pathology. The aim of this study was therefore to evaluate differences in OCT-estimated characteristics of nonculprit plaques between women and men with CAD.

## Methods

### Study population

**Fig. 1:**
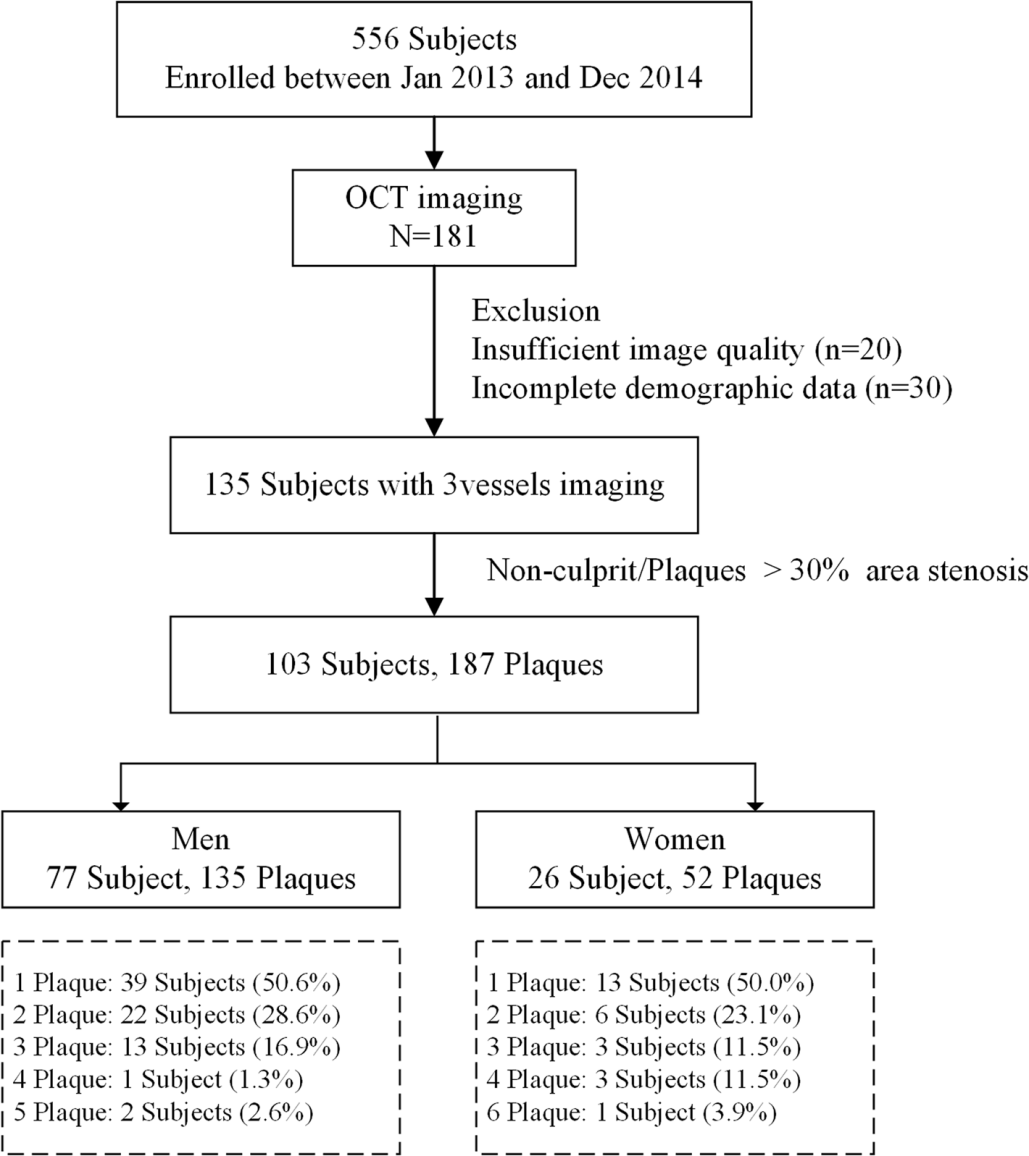
The patient count breakdown outline

Patient selection for the present study is summarized in Fig. 1. A total of 556 patients between January 2013 and December 2014 were retrospectively assessed, including 181 who had undergone OCT imaging. Only patients with complete information about clinical history, laboratory data, physical status, and sufficient image quality for all 3 vessels were selected. Therefore, 135 subjects with complete demographic data and sufficient 3-vessel OCT images were included. Plaques were identified with diameter stenosis > 30% by OCT compared with the reference vessel [19]. Patients without nonculprit plaques were also excluded. Based on angiograms, any plaque treated by percutaneous coronary intervention was excluded. Other exclusion criteria included myocardial infarction, cardiogenic shock, left main coronary artery disease, serious hepatic or renal dysfunction, massive thrombus and poor OCT image quality. Because it would be potentially difficult to acquire and interpret OCT-obtained images, patients with PCI, severe left ventricular dysfunction, or coronary artery bypass grafting were also excluded. Therefore, the final analysis included the remaining 103 subjects, with 187 nonculprit plaques.

This study was approved by the Ethics Review Committee of the Second Affiliated Hospital of Harbin Medical University, Harbin, China. All patients provided written informed consent.

### OCT image acquisition

All patients received standard antiplatelet and antithrombotic therapy with a loading dose of oral aspirin (300 mg) and ticagrelor (180 mg). Images were acquired on a commercially available frequency-domain OCT (FD-OCT) (C7XR system, Saint Jude Medical, Westford, MA, US). Intracoronary OCT examination was performed as previously described [20]. Briefly, a 2.7 F OCT imaging catheter (Dragonfly, LightLab Imaging Inc., Westford, MA) was advanced distal to the lesion, and automatic pullback was started as soon as the blood is cleared. All images were de-identified and digitally stored.

### Angiographic analysis

Coronary angiography was performed on a Cardiovascular Angiography Analysis System (CAAS 5.10, Pie Medical Imaging B.V., Maastricht, the Netherlands). The parameters of quantitative coronary angiography (QCA) included lesion length, reference vessel diameter (RVD), minimal lumen diameter (MLD) and diameter stenosis (DS). RVD was defined as the average diameter of the distal and proximal reference diameters. DS was derived as follow: DS = (RVD - MLD)/RVD × 100%.

### OCT image analysis

**Fig. 2:**
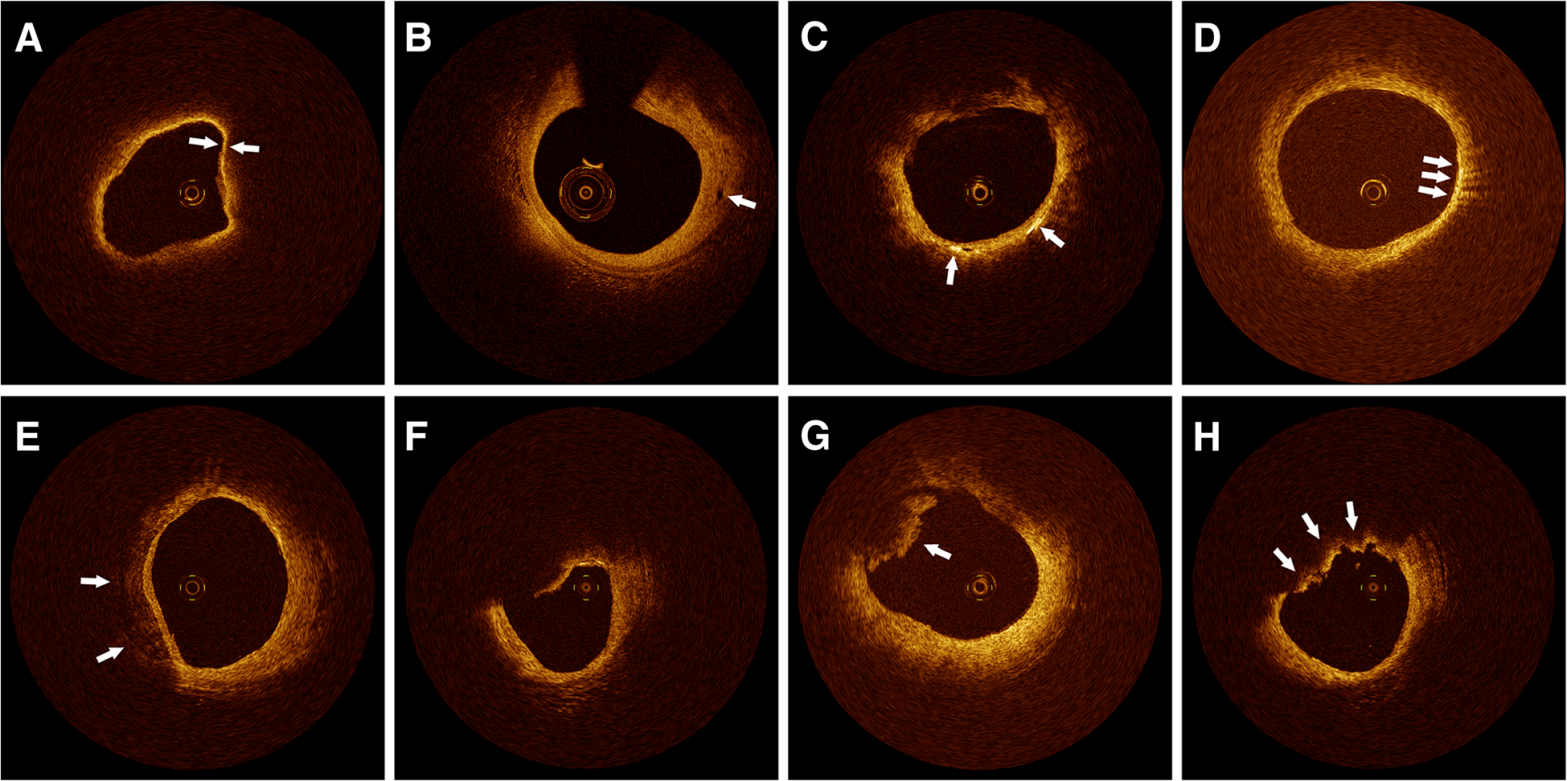
Representative optical coherence tomography images. **a** TCFA, defined as a lipid-rich plaque with a fibrous cap thickness < 65 μm (arrows). **b** Microvessels are represented by black holes within the plaque (arrow). **c** Cholesterol crystals are reflected by thin, linear regions of high intensity within the plaque (arrows). **d** Macrophage accumulation on OCT images, defined as increased signal intensity within the fibrous cap, accompanied by heterogeneous backward shadows (arrows). **e** Calcification is reflected by an area with low backscatter signal and a sharp border (arrows). **f** Plaque disruption showing discontinuity of the fibrous cap. **g** White (platelet-rich) thrombus is represented by low and homogeneous backscattering with low signal attenuation (arrow). **h** Red (red blood cell-rich) thrombus is represented by high backscattering with high attenuation (arrows)

Nonculprit plaques with diameter stenosis 20 to 70% on quantitative coronary angiography and culprit plaques undergoing percutaneous coronary intervention (PCI) were analyzed. A nonculprit plaque was defined as coronary artery stenosis with diameter stenosis 20 to 70%, for which PCI has not been performed. The acquired OCT images were analyzed by two investigators blinded to clinical presentations. In case of discordance between the two investigators, a consensus reading was obtained. Plaque characteristics were defined by previously validated criteria [21, 22]. A lipid-rich plaque was defined as one with lipid involving > 90° of the vessel wall circumference (lipid arc) [17]. For lipid-rich plaques, the lipid arc, lipid-core length, thinnest fibrous cap thickness (FCT) were determined, as well as the presence of thin-cap fibroatheroma (TCFA), macrophage accumulation, cholesterol crystals, and microvessels. Lipid-core length was defined as the length of plaque with > 90° of lipid arc and measured on the longitudinal view. The lipid index, defined as the mean lipid arc multiplied by lipid-core length [20], was also assessed. The fibrous cap thickness (FCT) of each lipid-rich plaque was measured 3 times at its thinnest part, and averaged. A thin cap fibroatheroma (TCFA) was defined as a lipid-rich plaque with a maximum lipid arc > 90° and a FCT < 65 μm [23] (Fig. 2a). A microvessel was defined as a signal-poor void without connection to the lumen, recognized on ≥3 consecutive cross-sectional images [22, 24] (Fig. 2b). Cholesterol crystals were defined as thin, linear regions of high intensity within the plaque [22, 25] (Fig. 2c). Macrophage accumulation on OCT images was identified by increased signal intensity within the plaque, accompanied by heterogeneous backward shadows [26, 27] (Fig. 2d). Calcification was also recorded when an area with low backscatter and a sharp border was identified inside the plaque [22] (Fig. 2e). Plaque disruption was defined as a discontinuity of the fibrous cap with communication between the vessel lumen and the cavity [23] (Fig. 2f). Thrombus was defined as a mass (diameter ≥ 250 μm) attached to the luminal surface or floating within the lumen [17, 22, 23] (Fig. 2g and h).

### Statistical analysis

All statistical analyses were performed with SPSS version 22.0 (IBM Corp., Armonk, NY). Categorical data were presented as number (percent) and compared by the χ^2^ or Fisher exact test, depending on data type. Continuous variables are mean ± standard deviation (SD); if necessary, median and 25th to 75th percentile were also presented. Differences between continuous variables were assessed by unpaired Student t test or Mann–Whitney test. *P* < 0.05 was considered statistically significant. For comparisons between groups, analysis was performed by means of the generalized estimating equations approach to take into account the within-subject correlation attributable to multiple plaques analyzed within a single subject. Univariate and multivariate linear regression analyses were performed to assess the relationship between lipid index and other factors.

## Results

### Patients and angiographic features

The baseline characteristics of the included patients are listed in Table 1. The 103 patients included 26 (25.2%) women and 77 (74.8%) men. Female patients were significantly older (mean age, 70.8 ± 7.3 vs 60.8 ± 9.8 years, respectively; *P* < 0.001) and had higher levels of low-density lipoprotein cholesterol (92.2 ± 27.0 vs 79.4 ± 27.6 mg/dL; *P* = 0.043) compared with men. Male patients were more likely to be current smokers (29.9% of men vs 3.8% of women; *P* = 0.007). No significant differences were found in the incidence rates of hypertension, diabetes mellitus, and drug use prior to admission between women and men.

**Table 1:** Baseline clinical characteristics

Variable	Men, *n* = 77	Women, *n* = 26	*P* Value
Age, y	60.8 ± 9.8	70.8 ± 7.3	< 0.001
Risk factors			
Hypertension, n (%)	47 (61.4)	13 (50.0)	0.324
Dyslipidemia, n (%)	39 (50.6)	15 (57.7)	0.534
Diabetes mellitus, n (%)	31 (40.3)	10 (38.5)	0.871
Current smoker, n (%)	23 (29.9)	1 (3.8)	0.007
Medications before admission			
Aspirin, n (%)	63 (81.8)	22 (84.6)	0.745
Statins, n (%)	57 (74.0)	21 (80.8)	0.488
β-Blockers, n (%)	29 (37.7)	10 (38.5)	0.942
ACE inhibitors, n (%)	19 (24.7)	7 (26.9)	0.820
Biochemistry data			
TC, mg/dL	151.6 ± 37.5	166.6 ± 37.5	0.082
LDL-C, mg/dL	79.4 ± 27.6	92.2 ± 27.0	0.043
HDL-C, mg/dL	40.2 ± 7.6	43.1 ± 12.6	0.164
TG, mg/dL	178.4 ± 167.7	149.9 ± 65.2	0.402
HbA_1c_, %	6.6 ± 1.3	6.9 ± 1.5	0.415
hs-CRP, mg/L	3.0 ± 2.8	1.6 ± 1.3	0.082
Creatinine, mg/L	0.9 ± 0.2	0.8 ± 0.2	0.018
Fasting glucose, mg/L	116.1 ± 41.8	139.1 ± 68.0	0.113

*Abbreviations: HbA*_*1c*_ glycated hemoglobin, *HDL-C* high-density lipoprotein cholesterol, *hs-CRP* high-sensitivity C-reactive protein, *IQR* interquartile range, *LDL-C* low-density lipoprotein cholesterol, *TC* total cholesterol, *TG* triglycerides. Data are n (%) or mean ± SD

Angiographic data are listed in Table 2. Reference vessel diameter (RVD), minimum lumen diameter (MLD) and diameter stenosis (DS) were not significantly different between male and female patients; however, lesion length was greater in men compared with women (9.4 ± 4.5 vs 7.3 ± 4.3, *P* = 0.024).

**Table 2:** Angiographic findings

Variable	Men, *n* = 135	Women, *n* = 52	*P* Value
Plaque location, n (%)			0.739
LAD, n (%)	45 (33.3)	15 (28.8)	
LCX, n (%)	37 (27.4)	17 (32.7)	
RCA, n (%)	53 (39.3)	20 (38.5)	
Multivessel disease, n (%)	38 (49.4)	13 (50.0)	0.954
QCA findings			
lesion length, mm	9.4 ± 4.5	7.3 ± 4.3	0.024
RVD, mm	3.0 ± 0.5	3.0 ± 0.6	0.640
MLD, mm	1.9 ± 0.4	1.9 ± 0.4	0.645
Diameter stenosis, %	37.3 ± 7.6	36.9 ± 7.7	0.750

*Abbreviations: LAD* left anterior descending artery, *LCX* left circumflex artery, *RCA* right coronary artery; *QCA, MLD* minimum lumen diameter, *RVD* reference vessel diameter, Data are n (%) or mean ± SD

### OCT findings

Quantitative and qualitative features of plaque morphologies by OCT are summarized in Table 3. No significant differences were found in minimum lumen area and mean reference vessel area between the two groups. Lipid-core length (9.54 ± 4.5 mm vs 7.3 ± 2.7 mm, *P* = 0.024) and lipid index (1615.1 ± 893.8 mm^2^ vs 1237.8 ± 859.8 mm^2^, *P* = 0.035) were significantly greater in men compared with women. There were no significant differences in FCT (men, 89.2 ± 34.9 μm; women, 93.4 ± 48.2 μm; *P* = 0.597) and TCFA prevalence (men, 37.7%; women, 46.2%; *P* = 0.444). The prevalence rates of other microstructures, such as disruption, calcification, microvessels, or cholesterol crystals, were not significantly different between genders.

**Table 3:** Optical coherence tomography (OCT) findings

Variables	Men, *n* = 77	Women, *n* = 26	*P* Value
MLA, mm^2^	3.1 ± 1.3	3.1 ± 1.3	0.746
Mean RVA, mm^2^	7.1 ± 2.5	7.3 ± 2.7	0.684
Stenosis area, %	42.6 ± 11.0	42.9 ± 10.8	0.880
Lipid-rich plaque			
Lipid-rich, n (%)	87 (64.4)	35 (67.3)	0.713
Maximum lipid arc	224.1 ± 63.6	210.0 ± 58.5	0.259
Lipid length, mm	9.4 ± 4.5	7.3 ± 4.3	0.024
Lipid index	1615.1 ± 893.8	1237.8 ± 859.8	0.035
FCT, μm	89.2 ± 34.9	93.4 ± 48.2	0.597
TCFA, n (%)	29 (37.7)	12 (46.2)	0.444
Disruption, n (%)	4 (3.0)	5 (9.6)	0.057
Calcification, n (%)	42 (31.1)	15 (28.8)	0.763
Macrophage, n (%)	42 (31.1)	17 (32.7)	0.835
Microvessels (MC), n (%)	50 (37.0)	27 (51.9)	0.064
Cholesterol crystal, n (%)	12 (8.9)	5 (9.6)	0.670
Thrombus, n (%)	2 (1.5)	3 (5.7)	0.103

*Abbreviations: FCT* fibrous cap thickness, *IQR* interquartile range, *MLA* minimum lumen area, *RVA* reference vessel area, *SD* standard deviation, *TCFA* thin-cap fibroatheroma. Data are n (%) or mean ± SD

### Univariate and multivariate linear regression models for Liped index

Table 4 shows the univariate and multivariate linear regression models. In the univariate model, sex and current smoker were all associated with a larger lipid index. Age, use of statin and aspirin were all associated with a smaller lipid index. The multivariate linear regression model demonstrated that only use of statin were an associated factor for a smaller lipid index after adjustment for other factors.

**Table 4:** Univariate and Multivariate Linear Regression Models for Lipid Index

	Univariate Model		Multivariate Model	
	β Coefficient	*P* Value	β Coefficient	*P* Value
Age	−21.012	0.004	−2.879	0.772
Sex	375.867	0.037	266.559	0.259
Hypertension	83.188	0.626	158.827	0.397
Dyslipidemia	221.820	0.458	552.927	0.097
Current smoker	469.748	0.014	163.004	0.474
Diabetes mellitus	304.668	0.070	230.157	0.171
Statins	− 644.889	0.000	− 783.199	0.023
β-Blockers	−226.489	0.171	9.594	0.961
Aspirin	−510.237	0.014	307.245	0.447
TC	−0.383	0.855	−0.688	0.889
LDL-C	3.453	0.200	1.754	0.769
HDL-C	−0.103	0.991	4.501	0.659
TG	−0.743	0.142	−0.617	0.486

*Abbreviations: HDL-C* high-density lipoprotein cholesterol, *LDL-C* low-density lipoprotein cholesterol, *TC* total cholesterol, *TG* triglycerides. *P* values are from the inference using the generalized estimating equation–based sandwich SE estimates

## Discussion

This OCT study focused on patients with CAD, and assessed gender differences in the characteristics of nonculprit plaques. We demonstrated that nonculprit plaques had reduced lipid index in female patients with CAD compared with male patients. The univariate linear regression model demonstrated that sex and current smoker were all associated with a larger LI, whereas in multivariate model only use of statin was independent risk factor for a larger LI.

At the time of presentation with CAD, women tend to be 10 years older than men [28]. Consistent with previously reported data, the present study found that women were significantly older when presenting with suspected CAD than men, with average ages of 70.8 and 60.8 years in women and men, respectively. It has been reported that the burden of coronary plaque increases with age [29], consistent with this result, univariate regression analysis in this study showed that age was positively correlated with LI. Even so, in present study, the LI of nonculprit plaques in man was still greater than that of plaque in female. In the present study, the active smoking rate of different gender patients was also different. The rate of current smokers in male patients was significantly higher than that of female patients. Previous angiographic and OCT studies shown that active smoking could increasing lipid accumulation [30, 31]. Our univariate regression analysis also showed that smoking was associated with greater LI, however, multivariate regression analysis showed that neither smoking nor gender was independent factor for LI. Therefore, smoking might contribute in part to the difference in nonculprit plaques between different genders.

Pathological studies have previously revealed differences in plaque morphologies between men and women [32–34]. A previous report demonstrated that among patients with ACS, women have less extensive coronary artery disease, by both angiographic and intravascular ultrasound (IVUS) measures, with lesions in women having less plaque rupture, reduced necrotic core and calcium, similar plaque burden, and smaller lumens compared with male patients, despite having more comorbid risk factors [35]. In this study, the LDL levels in female patients were significantly higher than those in male patients, however, the LI of female patients was smaller. Monique et al. [36] also used IVUS and near-infrared spectroscopy (NIRS) in CAD patients, and demonstrated that women have more favorable plaque characteristics than man, despite their worse risk profile.

On the other hand, several imaging studies have reported undifferentiated data regarding the relationship between gender and plaque characteristics. In patients with stable CAD, a multimodality intravascular imaging study observed no sex-specific differences in plaque morphology, as measured by lipid content, thin-cap fibroatheroma, calcification, microvessels, and macrophages in men and women [37]. Additionally, in patients with ACS, a previous study found no gender differences in the number of plaques with disruption, calcification, or thrombus [38]. Similarly, our team recently found that in patients with a first ST-segment elevation myocardial infarction (STEM), there are no differences in culprit plaque features between women and men [39]. In this study, nonculprit lesions in women with suspected CAD exhibited shorter lipid-cores and smaller LI compared with male counterparts. However, this did not translate into differences in fibrous cap thickness and TCFA frequency between genders. Whether this reflects differential effects of risk factor exposure on the artery wall in women requires further investigation. Gender-related differences were not observed in cholesterol crystals and calcification within nonculprit plaques in this study. The current findings are consistent with those of previous OCT studies [40] and complete previous reports assessing plaque features by OCT in CAD patients [39, 41].

The total number of deaths from cardiovascular diseases (CVD) is greater in women compared with men [28, 42]. However, OCT Imaging studies showed that plaque morphology in female CAD patients is not different from that of men, or even more stable compared with male plaques [39, 41, 42]. The reasons for gender-related differences in clinical outcomes remain unclear. A possible reason for higher mortality and morbidity in women might be the higher burden of risk factors compared with men, including higher prevalence rates of risk factors not included in traditional risk-estimation algorithms, such as depression, physical inactivity, and a family history of premature CAD [43]. Indeed, women are more affected by anxiety and depression than men [28], which may explain worse recovery in women, to a certain extent. Notably, the Women’s Ischemia Syndrome Evaluation study highlighted the importance of non-obstructive coronary artery disease, such as ischemia caused by microvascular disease, which is more commonly found in women compared with men [2, 44]. This phenomenon may partly explain why women have more favorable plaque characteristics, including reduced IL in nonculprit lesions, but show worse clinical outcomes. Long-term follow-up studies are required to comprehensively evaluate the clinical consequences of these differences.

### Study limitations

The limitations of the present study should be noted. First, this was a retrospective observational study from a database; therefore, selection bias may have influenced the above results. Secondly, adjustment for potential confounding factors was not performed because of the limited sample size. Thirdly, the study design had an intrinsic limitation related to the imbalance between women and men in the general population. Nonetheless, this imbalance reflects the true distribution of patients with CAD.

## Conclusions

In summary, nonculprit plaques contain reduced lipid cores in female patients with CAD compared with male counterparts. These findings highlight distinct pathophysiological features for atherosclerosis between genders, potentially leading to clinical presentation differences.

## Abbreviations

ACS: Acute coronary syndrome
CAAS: Cardiovascular Angiography Analysis System
CAD: Coronary artery disease
CVD: Cardiovascular disease
DS: Diameter stenosis
FCT: Fibrous cap thickness
IVUS: Intravascular ultrasound
LI: Lipid index
MLD: Minimal lumen diameter
OCT: Optical coherence tomography
PCI: Percutaneous coronary intervention
QCA: Quantitative coronary angiography
RVD: Reference vessel diameter
SD: Standard deviation
STEM: ST-segment elevation myocardial infarction
TCFA: Thin-cap fibroatheroma
